# Redefining Early Diabetic Kidney Disease Management: A Critical Review of Practice Patterns and Missed Opportunities in Real-World Settings

**DOI:** 10.7759/cureus.96931

**Published:** 2025-11-15

**Authors:** Abdullah Almalki

**Affiliations:** 1 Department of Medicine, King Abdullah International Medical Research Center (KAIMRC), Jeddah, SAU; 2 College of Medicine, King Saud Bin Abdulaziz University for Health Sciences College of Medicine, Jeddah, SAU; 3 Department of Nephrology, King Abdulaziz Medical City, Jeddah, SAU

**Keywords:** diabetic kidney disease (dkd), evidence-based clinical practice, microalbuminuria (ma), real-world data, screening program

## Abstract

Early diabetic kidney disease (DKD) is a silent yet progressive complication of diabetes and remains a leading cause of chronic kidney disease (CKD) and end-stage renal disease (ESRD) worldwide. Although international guidelines consistently recommend annual screening of urine albumin-to-creatinine ratio (UACR) and estimated glomerular filtration rate (eGFR), real-world adherence is persistently low. This narrative review synthesizes global and regional evidence - including findings from a cross-sectional study of more than 1,000 patients with diabetes - to highlight gaps between guideline recommendations and clinical practice. Deficiencies in screening are particularly evident in tertiary care settings, reflecting systemic barriers such as fragmented workflows, limited clinician awareness, and inadequate policy incentives. The expanding therapeutic landscape - including renin-angiotensin-aldosterone system (RAAS) inhibitors, sodium-glucose cotransporter-2 (SGLT2) inhibitors, and non-steroidal mineralocorticoid receptor antagonists (nsMRAs) - underscores the importance of early identification to optimize outcomes. Bridging this practice gap requires a coordinated, system-wide approach that integrates electronic health record (EHR) optimization, multidisciplinary collaboration, continuing professional education, and policy alignment to embed kidney health within comprehensive diabetes care. This review highlights the urgent need for implementation research and health-system reforms to achieve guideline-aligned DKD care worldwide.

## Introduction and background

Overview

Introduction

Diabetic kidney disease (DKD) is one of the most serious complications associated with diabetes mellitus, accounting for roughly 30%-40% of chronic kidney disease (CKD) cases worldwide and remaining a major contributor to end-stage renal disease (ESRD) [1,2]. According to the International Diabetes Federation (IDF) Diabetes Atlas 2024, an estimated 589 million adults worldwide are living with diabetes, which is projected to reach 853 million by 2050, underscoring the global scale of this epidemic [3]. In the Kingdom of Saudi Arabia and neighboring Gulf countries, the burden is especially severe due to the high prevalence of diabetes and lifestyle-related risk factors [4]. In its early stages, DKD often progresses without symptoms, with minor biochemical changes appearing before the clinical signs of kidney impairment. This asymptomatic phase presents a critical window for timely intervention to prevent long-term renal damage.

From a broader health standpoint, the consequences of DKD extend beyond renal deterioration. The condition is associated with increased cardiovascular risk, higher healthcare expenditures, and a greater likelihood of early mortality [5]. As such, early identification and treatment are key not only to slowing kidney disease but also to reducing cardiovascular complications and improving patient survival.

Routine screening for DKD typically involves two cost-effective and accessible assessments: the urine albumin-to-creatinine ratio (UACR) and estimated glomerular filtration rate (eGFR). These measurements are central to the early identification and risk classification of kidney involvement in diabetes and are strongly endorsed by recent international guidelines, including the KDIGO (Kidney Disease: Improving Global Outcomes) 2024 Clinical Practice Guideline for the Evaluation and Management of CKD and the American Diabetes Association's (ADA) Standards of Care in Diabetes-2024 [6,7].

Evidence-based treatments, including renin-angiotensin-aldosterone system (RAAS) inhibitors, sodium-glucose cotransporter 2 (SGLT2) inhibitors, and non-steroidal mineralocorticoid receptor antagonists (nsMRAs) like finerenone, have shown marked effectiveness in delaying CKD progression and lowering the incidence of cardiovascular events [8,9].

Despite the availability of these proven interventions, real-world adherence remains suboptimal. Data from recent surveys and patient registries reveal that fewer than half of individuals with diabetes receive annual testing for albuminuria - even in healthcare systems with advanced digital infrastructure [6,7]. This review combines both global research and regional insights, including data from the author’s recently published Cureus cross-sectional analysis [10], to examine the causes behind these practice gaps and to outline practical solutions for improving early DKD detection and management.

For this review, a structured literature search of PubMed, Scopus, and Google Scholar (January 2014 to June 2024) was performed using the terms “diabetic kidney disease,” “early detection,” “practice gap,” “implementation,” and “real-world management.” Priority was given to clinical trials, systematic reviews, and major international guidelines. Studies were included based on relevance to early detection, screening practices, and implementation strategies in diabetic kidney disease. As this review provides a qualitative synthesis, no quantitative pooling or meta-analysis was performed.

Pathophysiology and Rationale for Early Detection

The development of DKD results from a multifaceted set of disturbances that involve metabolic, hemodynamic, and inflammatory mechanisms acting together to damage the renal tissue. Sustained hyperglycemia triggers adaptive and maladaptive responses in the kidney, including elevated glomerular pressure and endothelial injury. Endothelial dysfunction and low-grade inflammation play pivotal roles in amplifying these effects, contributing to oxidative stress and progressive microvascular damage. These changes contribute to thickening of the glomerular basement membrane, expansion of mesangial cells, and excessive extracellular matrix accumulation [11]. Continuous intraglomerular hypertension leads to podocyte injury and loss, eventually promoting glomerulosclerosis - an early structural feature of DKD. Over time, these microvascular and structural abnormalities cause albumin leakage, decreased filtration capacity, and progressive nephron depletion.

Albuminuria represents one of the earliest detectable indicators of glomerular dysfunction and is also a reflection of systemic endothelial damage. An elevated UACR not only signals renal impairment but is also strongly associated with higher cardiovascular risk, even when the eGFR remains preserved [12,13]. This dual predictive value highlights albuminuria as an important therapeutic and prognostic marker for both renal and cardiovascular protection. However, recent data indicate that up to 40% of individuals with diabetes and a reduced eGFR show normal urinary albumin levels [14]. This normoalbuminuric pattern suggests alternative mechanisms, such as tubular, vascular, or interstitial injury rather than primary glomerular involvement, emphasizing the need for simultaneous UACR and eGFR assessment to achieve accurate risk classification.

Detecting DKD at an early stage provides an essential opportunity to modify disease progression. Early recognition allows clinicians to implement renoprotective interventions - such as RAAS blockade, SGLT2 inhibition, or non-steroidal mineralocorticoid receptor antagonists - while optimizing glycemic control, lipid profiles, and blood pressure management. When diagnosis is delayed, irreversible renal damage can develop, limiting treatment effectiveness and accelerating the transition to advanced CKD. Late detection also strains specialty care resources and increases healthcare expenditures. Thus, incorporating systematic DKD screening into routine diabetes care is a vital strategy for preserving kidney health and preventing long-term complications.

## Review

International guidelines versus real-world practice

The 2024 KDIGO Clinical Practice Guideline for CKD, the ADA Standards of Care in Diabetes, and the joint ADA-KDIGO 2022 Consensus Report all strongly recommend an annual assessment of UACR and eGFR in all adults with type 2 diabetes, starting at the time of diagnosis [6,7,15]. These guidelines emphasize risk stratification using the KDIGO “heatmap” model, which combines eGFR and albuminuria categories to guide clinical monitoring, treatment intensity, and referral thresholds. When applied consistently, this approach allows clinicians to intervene earlier, tailor therapy according to risk, and reduce progression to CKD and ESRD.

Despite strong supporting evidence and clear clinical protocols, integrating these recommendations into everyday practice continues to face obstacles. In the author’s Saudi-based cross-sectional study of over 1,000 adults with diabetes, only 49% underwent annual UACR screening, despite almost universal eGFR testing [10]. Interestingly, primary care centers showed better compliance than specialized endocrinology or general medicine clinics, indicating that the complexity of care settings does not always equate to better adherence to preventive measures. This observation points to a broader systems-level issue where guideline awareness may be high, but operational implementation remains inconsistent.

This gap is reflected internationally. In the United States, data from large healthcare systems reveal that just 30%-50% of eligible individuals with diabetes undergo recommended albuminuria testing each year [16]. In Europe, adherence varies between 40% and 70%, influenced by country-specific healthcare policies, reimbursement models, and the presence of automated reminder systems [17]. The shortfall is more apparent in several Asian and Middle Eastern nations, where rising diabetes prevalence is not yet matched by adequate infrastructure for integrated CKD screening programs [18]. In low- and middle-income countries, the lack of coordinated care between diabetes and kidney services and limited access to reliable laboratory testing remain major hurdles.

Several factors underlie this disconnect between recommendations and reality. Disjointed care delivery models, the absence of consistent clinical routines, and limited use of electronic decision-support tools all contribute to missed screening opportunities. In fast-paced outpatient environments, especially those managing large patient volumes, kidney function surveillance may be deprioritized. Additionally, ambiguity over whether primary care physicians, endocrinologists, or nephrologists are responsible for initiating UACR testing often leads to fragmented accountability. Collectively, these challenges suggest that improving practice adherence is less about clinical knowledge gaps and more about optimizing system design, workflow integration, and policy alignment. Addressing these barriers will require structural reforms that align healthcare delivery with evidence-based recommendations to improve population-level kidney outcomes.

Therapeutic advances and the expanding scope of early DKD care

Over the past decade, the therapeutic landscape of DKD has undergone a major transformation, shifting from single-pathway blockade to multidrug, multimodal protection. Beyond traditional RAAS inhibition, new pharmacologic agents such as sodium-glucose cotransporter-2 (SGLT2) inhibitors and non-steroidal mineralocorticoid receptor antagonists (nsMRAs) have redefined the standard of care by targeting complementary metabolic and hemodynamic pathways involved in kidney injury.

SGLT2 inhibitors - such as dapagliflozin, canagliflozin, and empagliflozin - have demonstrated consistent renal and cardiovascular benefits in patients with type 2 diabetes, as evidenced by the Dapagliflozin and Prevention of Adverse Outcomes in Chronic Kidney Disease (DAPA-CKD), Canagliflozin and Renal Events in Diabetes with Established Nephropathy Clinical Evaluation (CREDENCE), and Study of Heart and Kidney Protection with Empagliflozin (EMPA-KIDNEY) trials, as well as a comprehensive meta-analysis of cardiovascular outcome studies [8,19-21]. These agents improve intraglomerular pressure regulation, reduce albuminuria, preserve kidney function, and lower the risk of kidney failure and cardiovascular mortality. Their effects extend beyond glycemic control, establishing SGLT2 inhibitors as disease-modifying therapies that address the multifactorial mechanisms of diabetic nephropathy and cardiorenal dysfunction.

Non-steroidal MRAs, with finerenone as a leading agent, offer additional therapeutic value. Findings from the Finerenone in Reducing Kidney Failure and Disease Progression in Diabetic Kidney Disease (FIDELIO-DKD) and Finerenone in Reducing Cardiovascular Mortality and Morbidity in Diabetic Kidney Disease (FIGARO-DKD) trials revealed that finerenone, when used alongside optimized RAAS inhibition, significantly delayed CKD progression and reduced cardiovascular risk [9,22,23]. Its mechanism includes anti-inflammatory and antifibrotic actions, with a lower tendency to induce hyperkalemia compared to steroidal MRAs. This positions finerenone as a complementary agent to SGLT2 inhibitors within a multifaceted strategy aimed at preserving renal and cardiovascular function.

Collectively, these therapies represent a shift in early DKD care from reactive management toward a more proactive, protective approach. Nevertheless, despite strong evidence from randomized trials, their integration into real-world clinical settings remains limited. Observational data suggest that a substantial number of eligible patients do not receive these treatments due to factors such as therapeutic inertia, affordability issues, formulary restrictions, and limited awareness among general practitioners and non-specialists [24]. To overcome these obstacles, health systems must prioritize clinician education and streamline care pathways, ensuring that early detection via UACR and eGFR testing leads to prompt and appropriate initiation of renoprotective therapies. Narrowing the gap between clinical trial efficacy and everyday clinical use is essential to realizing the full potential of these advances.

Systemic barriers and missed opportunities

The gap between evidence-based guidelines and real-world practice arises from a multifaceted interplay of provider, system, and policy-level barriers that operate across the continuum of care. At the provider level, awareness and prioritization of DKD screening remain inconsistent. Many non-nephrology physicians - particularly internists, diabetologists, and cardiologists - tend to focus primarily on glycemic and lipid management, often overlooking renal surveillance. This lack of emphasis is partly due to the misperception that albuminuria testing falls under another specialist’s domain. As a result, screening may be deferred or omitted entirely, especially when patients appear clinically stable or when consultations are time-constrained [25]. Even when providers are familiar with the recommendations, DKD screening is sometimes seen as secondary to more immediate clinical demands, reflecting a gap not of knowledge but of workflow integration.

Challenges within clinical workflows and operational routines further contribute to the low uptake of albuminuria testing. In many outpatient settings, default lab panels for diabetes management routinely include glycated hemoglobin (HbA1c) and lipid levels, while omitting UACR. As clinicians often rely on pre-set order sets, this omission results in missed testing unless actively corrected. Additionally, logistical barriers - such as requiring a separate visit to provide a urine sample - reduce patient compliance. Integrating UACR into standard diabetes panels and enabling nurse- or technician-initiated testing could simplify processes and improve uptake, but such strategies are inconsistently applied. Differences in institutional workflow design and resource availability significantly influence whether best practices are implemented and maintained.

The role of digital systems is also critical. Electronic health records (EHRs), when effectively configured, can support preventive care by prompting providers at the point of decision-making. For example, an implementation study within an integrated U.S. health system demonstrated that nephrology-specific electronic consultation pathways and targeted alerts improved CKD management and facilitated more timely referrals [26]. Similar improvements have been reported in primary care settings internationally, where automated reminder systems embedded within EHR platforms substantially increased UACR screening rates [17,27]. However, widespread adoption remains uneven. In some systems, limited technological capacity hinders the implementation of tailored alerts. In others, excessive prompts lead to “alert fatigue,” causing clinicians to disregard even relevant reminders. Optimizing EHRs to be intuitive, adaptable, and well-integrated into clinical routines is vital to enhance their effectiveness.

Financial and policy-related obstacles also influence DKD screening practices. In many healthcare environments, routine renal monitoring is not directly tied to reimbursement models, quality metrics, or performance incentives. This absence of structured accountability reduces institutional urgency to prioritize kidney screening. On the other hand, when renal assessments are embedded in quality dashboards or linked to organizational goals, screening rates improve significantly, highlighting the role that health policy and payment systems can play in shaping clinical behavior [28].

Lastly, low patient and public awareness presents another significant hurdle. Because early DKD progresses silently, many individuals are unaware of its risks and do not recognize the importance of regular screening. Public awareness campaigns - such as community outreach or mobile health messaging - have shown encouraging results in improving knowledge and promoting participation in screening programs [29]. Embedding kidney health messages within standard diabetes education curricula can empower patients to actively seek timely testing. Ultimately, closing the DKD implementation gap will require coordinated efforts to tackle interrelated provider, system, and patient-level barriers through sustained, system-wide strategies.

Strategies to bridge the implementation gap

Closing the DKD care gap necessitates a multifaceted and coordinated approach that spans digital innovation, professional education, workflow redesign, multidisciplinary collaboration, and supportive policy frameworks. These domains are interdependent and must operate synergistically to transform evidence into practice effectively.

Digital-Level Innovations

Leveraging digital infrastructure is a key driver of improved screening uptake. Incorporating automated prompts for overdue UACR and eGFR testing into EHR systems, integrating them with laboratory ordering workflows, and utilizing feedback dashboards have all demonstrated success in improving adherence. For example, healthcare organizations such as Kaiser Permanente in the United States and national programs within the UK’s National Health Service (NHS) have reported substantial gains - nearly doubling albuminuria screening rates - after implementing these digital interventions [16,17]. In parallel, emerging applications of artificial intelligence (AI) and machine-learning-based predictive models are increasingly being used to identify high-risk individuals, allowing for earlier intervention and nephrology referral before irreversible kidney damage develops [30].

Provider-Level Strategies

Enhancing professional education and accountability is equally important. Continuing medical education (CME) should go beyond didactic sessions and include practical training focused on real-world application - such as interpreting urine albumin trends, recognizing thresholds for treatment initiation, and incorporating new therapies into individualized care plans. Promoting consistent uptake of updated KDIGO and ADA guidance across both primary and specialty care helps ensure a unified standard of care. In addition, linking DKD screening performance metrics to clinician evaluations or institutional quality benchmarks can strengthen accountability and help translate educational initiatives into sustained improvements in daily practice [24,31].

Workflow-Level Interventions

Improving workflows involves embedding evidence-based practices into routine clinical operations. Including UACR and eGFR in standard diabetes visit templates can help ensure screening is not overlooked. Nurse-led testing protocols and assigning care coordinators to track follow-up on abnormal results can enhance continuity of care. Establishing regular audit-and-feedback cycles that assess clinician adherence and compare performance across clinics promotes accountability, supports ongoing quality improvement, and drives consistent implementation of DKD screening standards.

Patient- and Team-Level Approaches

Team-based care models have also been shown to improve outcomes. Coordinated involvement of endocrinologists, nephrologists, diabetes educators, and case managers fosters shared responsibility and reduces fragmentation of care. Studies have shown that multidisciplinary approaches not only slow kidney disease progression but also improve patient satisfaction and reduce hospitalization rates [31]. Effective collaboration is further supported by shared access to EHRs and clear communication pathways between care teams, allowing for prompt action when concerning findings arise. At the patient level, structured education, empowerment strategies, and self-management support play a crucial role in sustaining adherence, optimizing lifestyle factors, and maintaining long-term kidney health.

Policy-Level Frameworks

Policy-level action provides the structural foundation for long-term change. Establishing albuminuria screening as a recognized quality measure - whether at the organizational or national level - aligns provider efforts with public health goals and increases accountability. Countries that have embedded DKD indicators into broader chronic disease frameworks have reported marked improvements in early diagnosis and therapy uptake [32]. Additional strategies such as linking adherence to reimbursement models, public reporting requirements, or accreditation standards may help reinforce and sustain these gains.

Ultimately, closing the DKD care gap requires a systemic, not just individual, response. Effective management hinges on alignment across digital tools, clinical training, operational design, and policy direction. Only through an integrated, system-wide approach can health systems shift from reactive treatment to proactive kidney care.

Future directions

The future of early DKD management is being shaped by advances in precision medicine, digital innovation, and system-level integration. Emerging biomarkers - such as soluble urokinase plasminogen activator receptor (suPAR), kidney injury molecule-1 (KIM-1), neutrophil gelatinase-associated lipocalin (NGAL), and novel metabolomic or proteomic signatures - are expected to refine risk prediction and improve phenotypic characterization beyond traditional markers like the UACR and eGFR [33]. These molecular tools may enable earlier identification of high-risk individuals, guide tailored therapeutic interventions, and serve as surrogate endpoints in clinical trials aimed at halting disease progression before irreversible nephron loss occurs. However, their integration into clinical practice remains constrained by limited large-scale validation, heterogeneity in assay performance, and uncertain cost-effectiveness, emphasizing the need for standardized evaluation and health-economic modeling before widespread implementation.

Digital health technologies will likely play a pivotal role in transforming DKD detection and monitoring. Smartphone-based urine dipstick analyzers, remote laboratory connectivity, and telehealth platforms can decentralize screening and make kidney health surveillance accessible to patients in both urban and remote settings. Artificial intelligence (AI)-assisted algorithms integrated into EHRs have already demonstrated potential in predicting CKD progression, optimizing testing intervals, and supporting clinical decision-making. These data-driven approaches can bridge geographic and socioeconomic barriers, empowering patients to participate actively in disease prevention through self-monitoring and digital engagement.

At the system and policy levels, the coming decade should see greater integration of kidney health into national chronic disease frameworks. Establishing centralized kidney registries that track DKD screening, therapy initiation, and long-term outcomes will generate actionable real-world evidence to inform health planning. Linking performance metrics - such as annual UACR completion rates - to institutional quality reporting and bundled reimbursement schemes may incentivize adherence to evidence-based care. Furthermore, global collaborations across nephrology societies, diabetes federations, and digital health stakeholders can standardize data sharing, benchmarking, and innovation pipelines.

Ultimately, the convergence of precision biomarkers, digital analytics, and coordinated health policy provides an unprecedented opportunity to redefine DKD prevention and management. By leveraging technology and cross-sector collaboration, healthcare systems can move beyond reactive treatment paradigms toward anticipatory, patient-centered kidney care that aligns with the future of precision medicine.

## Conclusions

Bridging the persistent gap between evidence and real-world practice in early DKD management is both an attainable and necessary goal with broad clinical and public health implications. Although the prognostic and cost-effective value of routine UACR and eGFR testing is well established, adherence to these essential practices remains suboptimal in most healthcare settings. This inconsistency reflects not a lack of knowledge but a systemic challenge involving fragmented workflows, insufficient provider incentives, and limited integration of kidney health within broader diabetes care frameworks.

Achieving meaningful improvement requires a coordinated and sustained response across multiple levels of the healthcare system. Integrating digital solutions - such as automated EHR reminders, point-of-care prompts, and feedback dashboards - can embed screening into routine clinical behavior. These tools should be complemented by continuous professional education that reinforces the centrality of DKD screening and ensures physicians, nurses, and allied health professionals remain aligned with evolving guideline recommendations. In parallel, fostering team-based and multidisciplinary care models can ensure seamless communication between endocrinologists, nephrologists, and primary care providers, thereby reducing delays in detection and treatment.

From a policy perspective, embedding DKD screening within national and institutional quality metrics provides structural reinforcement for sustained adherence. Linking screening performance to quality reporting, accreditation, or reimbursement mechanisms has demonstrated positive outcomes in other chronic disease domains and may serve as a model for kidney health. Such policy-driven approaches should also emphasize equity, ensuring that patients across diverse healthcare environments, including low-resource settings, can benefit from early detection and timely intervention.

*Key Actionable Recommendations*: Health systems should (1) integrate automated UACR and eGFR alerts into diabetes care dashboards, (2) establish reflex nephrology referral pathways for high-risk patients, (3) embed evidence-based use of SGLT2 inhibitors and finerenone into standardized care protocols, and (4) link DKD screening adherence to institutional quality metrics and reimbursement frameworks. Collectively, these actions can operationalize early detection and ensure timely initiation of renal-protective therapies.

Ultimately, improving early DKD detection and management not only delays progression to ESRD but also substantially reduces cardiovascular morbidity, mortality, and healthcare expenditures. As the prevalence of diabetes continues to rise worldwide, the need for proactive, data-driven kidney care becomes more urgent. The convergence of novel pharmacotherapies, digital analytics, and public health reform provides an unprecedented opportunity to transform DKD management from reactive treatment to true prevention. Embedding DKD screening within chronic disease management dashboards and linking detection to timely initiation of renal-protective therapy can help close the persistent evidence-to-practice gap and reduce the burden of DKD progression in real-world care.
